# LAPAROSCOPIC ANTIREFLUX SURGERY: ARE OLD QUESTIONS ANSWERED? SHOULD IT BE USED CONJOINED WITH ENDOSCOPIC THERAPY FOR BARRETT’S ESOPHAGUS?

**DOI:** 10.1590/0102-672020210002e1664

**Published:** 2022-06-24

**Authors:** Shiwei HAN, Donald E. LOW

**Affiliations:** 1 Department of General and Thoracic Surgery, Virginia Mason Medical Center, Seattle, WA, USA.

**Keywords:** Gastroesophageal Reflux, Fundoplication, Laparoscopy, Barrett’s Esophagus, Refluxo Gastroesofágico, Fundoplicatura, Laparoscopia, Esôfago de Barrett

## Relationship of Barrett’s esophagus, gastroesophageal reflux disease, and esophageal adenocarcinoma

Barrett’s esophagus (BE) represents the morphological premalignant manifestation of gastroesophageal reflux disease (GERD), which develops as a consequence of the dysfunction and failure of the antireflux mechanism^38^. BE involves the formation of intestinal metaplasia (IM) from the squamous epithelium of the esophagus, which is a reparative response to reflux-induced damage^37^. Although the prevalence in Western countries is about 1-2% in the general population and about 10% in population who report acid reflux symptoms, the accurate prevalence of BE in the general population is difficult to determine as the majority of individuals with BE are not diagnosed^40^ ^,^ ^42^. Epidemiological and histopathological evidence indicate that many cases of esophageal adenocarcinoma (EAC) arise in individuals with BE by the progression of IM (nondysplastic Barrett’s esophagus [NDBE]) and indefinite for dysplasia (IND) to dysplasia (including low-grade dysplasia [LGD] and high-grade dysplasia [HGD]) and finally to neoplasia^46^. To date, dysplasia remains the best available marker of cancer risk in patients with BE.

Since BE is considered a complication of chronic GERD, it is perhaps not surprising that risk factors for gastric reflux are also strongly associated with BE^30^ ^,^ ^54^. Reflux-induced injury has been linked to cellular and molecular changes in the esophagus^12^ ^,^ ^39^. Symptoms of heartburn and regurgitation are strongly associated with the presence of BE, and duration of GERD symptoms may also be a risk factor for BE^7^. Although GERD is a strong risk factor for both BE and EAC, 40-50% of patients with these disorders do not report chronic reflux symptoms, suggesting that silent reflux or other risk factors such as male sex^57^, age 50 or older^43^, white race^58^, central obesity^28^, and cigarette smoking^9^ also likely play a role in the pathogenesis of BE and EAC.

Although BE is well-established precursor for EAC, the assumption that all patients who develop EAC go through the same reflux-induced response leading to adenocarcinoma was challenged by a retrospective analysis that found that only 46% of patients with EAC presented with endoscopic confirmation of BE and histopathological evidence of IM^45^. Furthermore, comparison of patients with EAC who had confirmed BE at presentation to those without BE suggested the existence of two EAC phenotypes with different tumor behavior and response to therapy^45^. These findings raise the question of whether EAC always develops through the IM-dysplasia-EAC sequence.

## Current management of Barrett’s esophagus

Accepting that a controversy exists, the natural course of progression to dysplasia and cancer in BE in the majority of patients is thought to be stepwise from NDBE to LGD to HGD and cancer. The annual cancer risk depends on the degree of dysplasia, such as 0.33% if there is no dysplasia, 0.54% with LGD, and 7% with HGD^47^. Thus, the management is based on disease stages.

###  1. Nondysplastic Barrett’s esophagus 

Proton-pump inhibitor (PPI) therapy is recommended to control reflux symptoms in patients with NDBE. The American College of Gastroenterology (ACG)^47^, American Gastroenterological Association (AGA)^52^, and American Society for Gastrointestinal Endoscopy (ASGE)^46^ all recommend that surveillance endoscopy with four-quadrant biopsies at 2-cm intervals every 3-5 years for NDBE. PPI therapy is associated with a 71% decrease in the risk of developing HGD and EAC in patients with BE^50^. Long-term therapy (>2-3 years) has a higher protective effect^50^. Chemoprevention to inhibit the progression to cancer in patients with BE is currently being assessed. Various medications such as aspirin, metformin, and statins have been studied. A randomized controlled trial indicated that the combination of high-dose esomeprazole plus aspirin had the strongest protective effect compared with low-dose esomeprazole without aspirin at a median follow-up of 8.9 years^25^. However, the ACG guidelines do not currently recommend chemoprevention for all patients with BE, but suggest it should be considered in patients with BE who are appropriate candidates for aspirin use for cardioprotection^47^.

###  2. Indefinite for dysplasia 

In BE IND, either the epithelial abnormalities are insufficient for a diagnosis of dysplasia, or the nature of the epithelial abnormalities is uncertain due to inflammation or technical difficulties with specimen processing. The risk of HGD or cancer within 1 year of the diagnosis of IND varies between 1.9% and 15%^55^. The recommendation from ACG^47^ for management is to optimize acid suppressive therapy for 3-6 months and then to repeat esophagogastroduodenoscopy (EGD). If indefinite dysplasia is noted again, repeat endoscopy in 12 months is recommended^59^.

###  3. Low-grade dysplasia 

Most patients with an initial diagnosis of LGD (73%) are downstaged to NDBE or to IND after review by expert gastrointestinal pathologists^10^. Patients with confirmed and persistent LGD are at higher risk of progression^11^. Once LGD is confirmed by a second gastrointestinal pathologist, the patient should be considered for endoscopic ablation. A landmark study demonstrated the benefit of radiofrequency ablation in achieving complete eradication of dysplasia (90.5% vs. 22.7% for a sham procedure) and complete eradication of IM (77.4% vs. 2.3% for a sham procedure)^49^. Patients with confirmed LGD who do not undergo eradication therapy should have surveillance endoscopy every 6-12 months.

###  4. High-grade dysplasia 

As with LGD, the diagnosis of HGD needs to be confirmed by a second pathologist with gastrointestinal expertise. In the past, the treatment was esophagectomy, but due to demonstrated lower morbidity and equivalent efficacy of radiofrequency ablation, the current treatment of choice is endoscopic mucosal resection (EMR) of raised lesions, followed by radiofrequency ablation of the entire affected segment^23^. Pathology is best assessed by EMR, especially in areas of nodularity and ulceration. A randomized controlled study of 42 patients with HGD was randomized between radiofrequency ablation and sham procedure. Complete eradication of dysplasia was achieved in 81% of ablation patients versus 19% with the sham procedure^49^. Eradication of IM was achieved in 77% of ablation patients versus 2% of patients with the sham therapy^49^. Results of 3-year follow-up from the same cohort showed complete eradication of dysplasia in 98% and of IM in 91%^48^. Endoscopic eradication therapy is recommended for all patients with BE and HGD without the potential comorbidity and side effects associated with esophageal resection. Short segment Barrett’s (<3 cm) with HGD can also be assessed for complete ablation with EMR alone. Alternatively, surveillance every 3 months is an option if the patient does not wish to undergo eradication therapy^48^.

## What is the role of antireflux surgery in the treatment of Barrett’s esophagus?

Because dysplasia in BE carries an increased risk of progression to cancer, the current standard of care in these patients is EMR of visible lesions, followed by ablation of the flat mucosa, with the aim of achieving complete eradication of IM^7^ ^,^ ^47^. A key part of treatment during this time is maximal acid suppression with continuous PPI treatment^16^.

PPIs are today the main component of medical treatment for GERD, because they are the most effective medications to decrease gastric acid production, leading to healing of esophagitis and relief of symptoms^31^. However, PPIs only change the pH of the refluxate, without modifying the occurrence and the number of reflux episodes^53^. Therefore, symptoms tend to recur after discontinuation of PPIs, and some patients on PPIs have refractory symptoms due to ongoing reflux.

Successful elimination of reflux symptoms does not guarantee control of acid reflux. Often, BE patients do not experience heartburn due to the reduced sensitivity of the columnar mucosa to the acidic and bilious refluxate^21^. In addition, while PPI stops acid reflux, patients may still have regurgitation as a consequence of an incompetent lower esophageal sphincter (LES)^32^ and the quality of esophageal peristalsis^33^. Potentially most significantly, PPIs do not eliminate the reflux of bile, a major contributor to the pathogenesis of BE^6^ ^,^ ^13^ ^,^ ^26^.

As many as 40% of patients with heartburn have either an incomplete or complete lack of response to once-daily PPIs^22^. The proportion of patients with persistent troublesome heartburn despite once-daily PPI use was 32% in randomized trials and 17% in nonrandomized trials; the proportion of patients with persistent regurgitation was 28% in randomized and nonrandomized trials^14^. In addition, increasing evidence has highlighted the risk of adverse events and side effects after long-term PPI treatment, including kidney disease and injury^2^, *Clostridium difficile* infection^17^, community-acquired pneumonia^15^, fractures due to osteoporosis^24^, gastric cancer^8^, and increased risk of COVID-19^4^.

Antireflux surgery (ARS) aims to repair the antireflux barrier, which is defective in patients with BE^34^. As a result, the function of the LES is improved, the gastroesophageal flap valve is restored, and acid or duodenal reflux into the esophagus is decreased compared to medical treatment alone^44^. The most common indication for ARS is refractory symptoms or persistent esophagitis that is not responding to medical therapy^1^. The most commonly performed surgical procedure for GERD is laparoscopic fundoplication, which enhances the esophagogastric junction ability to prevent reflux into the esophagus^19^. A 5-year follow-up of 372 patients included in an randomized control trial comparing the PPI esomeprazole with laparoscopic fundoplication found similar remission rates in the medication group (92%; 95%CI 89-96%) and surgery group (85%; 95%CI 81-90%), but worse symptoms of acid regurgitation in the medication group (13%) compared with the surgery group (2%)^18^. A few studies have compared the effect of ARS on BE with best medical therapy and indicated that the surgery intervention had significantly less dysplasia de novo^35^ and a greater probability of BE regression^33^. Laparoscopic fundoplication is safe and has been associated with a very low short-term mortality (0.1-0.2%). Complications and, more importantly, side effects of gas bloat and inability to belch and vomit can occur and should be a component of counseling and discussion with the patient preoperatively^61^.

Other indications for ARS in patients with BE include younger patients who do not wish to commit themselves to lifelong PPI therapy. It is particularly important that these patients are counseled that the surgery is being considered to control their GERD and decrease their reliance on PPIs, not to eliminate the requirement for long-term endoscopic Barrett’s follow-up^19^.

The optimum timing of ARS in patients with Barrett’s has not been standardized. Some reports have suggested that performing ARS at the time of ablative therapy can decrease the number and improve the efficiency of endoscopic Barrett’s ablation^20^. Failure of laparoscopic fundoplication can often be linked to applying incorrect indications of inadequate preoperative assessment^36^. As a result, accurate preoperative assessment including endoscopy, high-resolution manometry, and selective application of objective pH testing to define those who will benefit from the ARS, i.e., those with LES dysfunction and strong symptom correlation, is recommended. There are recognized advantages of having the procedures conducted in centers with high volume experience and with the capability of delivering the full spectrum of diagnostic workup, surgical treatment, and follow-up of GERD and BE^41^.

Controversies are still present regarding the progression of Barrett’s following ARS. One study based on a Swedish population showed that ARS failed to prevent the development of esophageal cancer when compared with the corresponding population^29^. Other studies have suggested that successful ARS protects against progression to malignancy; however, this has not been confirmed in prospective trials or large cohort studies^30^ ^,^ ^56^ ^,^ ^60^.

Increasingly, patients with LGD are undergoing successful endoscopic ablation^27^. Patients with LGD can be considered for ARS^51^, and recent reports suggest that fundoplication is superior to medical therapy in avoiding Barrett’s progression and promoting Barrett’s regression^60^. There are currently very few data on whether successful ARS decreases the incidence of recurrence following successful ablation of either LGD or HGD.

Antireflux surgery can be considered in patients following successful ablation of HGD. However, many surgeons would advocate an extended period of stable postablation endoscopic follow-up before proceeding with ARS. Some practitioners recommend repeating objective pH testing following ARS in patients with NDBE as well as those with LGD or HGD postablation^3^. Whether this testing should be done before medical therapy is discontinued has not been extensively studied^3^.

## CONCLUSION

The current recommended BE treatment is maximal acid suppression with PPIs and histamine-2 blockers, while in some cases, fundoplication is required to control reflux refractory to medical therapy. In our opinion, laparoscopic ARS can be an appropriate alternative and even a preferred option from medical therapy for highly selected candidates. Minimal morbidity and near zero mortality in high volume centers along with multiple studies demonstrating long-term success of antireflux operations support this approach. However, post-treatment surveillance continues to be a required component of long-term treatment because the risk of progression to dysplasia still exists. Nevertheless, a prospective randomized controlled trial is needed to confirm the therapeutic effect and long-term outcomes of laparoscopic ARS versus medical therapy plus or minus endoscopic ablation in patients with BE. In addition, future comparisons of maximal medical therapy versus other surgical techniques (LINX device) and endoscopic antireflux procedures such as TIF (Transoral Incisionless Fundoplication) and Stretta are also warranted.
